# A Facile Method for the Non-Covalent Amine Functionalization of Carbon-Based Surfaces for Use in Biosensor Development

**DOI:** 10.3390/nano10091808

**Published:** 2020-09-10

**Authors:** Ffion Walters, Muhammad Munem Ali, Gregory Burwell, Sergiy Rozhko, Zari Tehrani, Ehsaneh Daghigh Ahmadi, Jon E. Evans, Hina Y. Abbasi, Ryan Bigham, Jacob John Mitchell, Olga Kazakova, Anitha Devadoss, Owen J. Guy

**Affiliations:** 1 Centre for NanoHealth, College of Engineering, Swansea University, Swansea SA2 8PP, UK; 823439@swansea.ac.uk (M.M.A.); z.tehrani@swansea.ac.uk (Z.T.); e.daghighahmadi@swansea.ac.uk (E.D.A.); j.e.evans@swansea.ac.uk (J.E.E.); h.y.abbasi@swansea.ac.uk (H.Y.A.); j.j.mitchell@swansea.ac.uk (J.J.M.); 2 Department of Physics, College of Science, Swansea University, Swansea SA2 8PP, UK; g.burwell@swansea.ac.uk (G.B.); r.m.bigham@swansea.ac.uk (R.B.); 3 National Physical Laboratory, Quantum Metrology Institute, Teddington, Middlesex TW11 0LW, UK; sergiy.rozhko@npl.co.uk (S.R.); olga.kazakova@npl.co.uk (O.K.); 4 Department of Chemistry, College of Science, Swansea University, Swansea SA2 8PP, UK

**Keywords:** graphene, non-covalent, biosensor, real-time, sensor, nanocomposite, π-π stacking, drop-cast, carbon-surfaces, resistor, GFET

## Abstract

Affinity biosensors based on graphene field-effect transistor (GFET) or resistor designs require the utilization of graphene’s exceptional electrical properties. Therefore, it is critical when designing these sensors, that the electrical properties of graphene are maintained throughout the functionalization process. To that end, non-covalent functionalization may be preferred over covalent modification. Drop-cast 1,5-diaminonaphthalene (DAN) was investigated as a quick and simple method for the non-covalent amine functionalization of carbon-based surfaces such as graphene, for use in biosensor development. In this work, multiple graphene surfaces were functionalized with DAN via a drop-cast method, leading to amine moieties, available for subsequent attachment to receptor molecules. Successful modification of graphene with DAN via a drop-cast method was confirmed using X-ray photoelectron spectroscopy (XPS), Raman spectroscopy and real-time resistance measurements. Successful attachment of receptor molecules also confirmed using the aforementioned techniques. Furthermore, an investigation into the effect of sequential wash steps which are required in biosensor manufacture, on the presence of the DAN layer, confirmed that the functional layer was not removed, even after multiple solvent exposures. Drop-cast DAN is thus, a viable fast and robust method for the amine functionalization of graphene surfaces for use in biosensor development.

## 1. Introduction

Graphene possesses exceptional electrical properties [1,2] such as extremely high carrier mobility, high electron transfer rates, gate tenability [3] and highly efficient fluorescence quenching, making graphene an attractive platform for electrical, electrochemical, and optical sensor technologies [4]. Affinity biosensor manufacture requires bio-functionalization of the graphene surface with an analyte-specific receptor [5]. Adsorption of proteins to a graphene surface for use in biosensors requires the protein to remain in a particular conformation, ensuring one part of the protein is in contact with the graphene surface (required for adsorption stability and electron doping) and another specific part is exposed to the solvent/solution phase (required for receptor-analyte binding) [6,7]. Protein adsorption to a surface is a complicated process involving van der Waals, hydrophobic, and electrostatic interactions, and hydrogen bonding [8], with process conditions, such as ionic strength, pH, and temperature also contributing factors [9]. Non-covalent interactions between proteins and graphene depend on the binding affinity of various residues in the protein as well as the distribution of the residues [6] and it has not been explicitly shown that a protein remains functional after adsorption onto graphene surfaces [10]. Therefore, different unique proteins could interact differently with the graphene surface, potentially affecting the proteins’ structure and binding capability. Surface modification strategies are necessary to ensure the binding of proteins to preferential sites and to help maintain protein conformation. To that end, chemical functionalization of the graphene surface before attachment of receptor proteins is essential to the development of working biosensors.

The functionalization of carbon surfaces can be either covalent [11] or non-covalent [12]. Covalent functionalization involves reaction with the sp^2^ carbon bonds in the aromatic lattice and the subsequent introduction of sp^3^ bonds at these reaction sites, reducing the aromaticity of the graphene lattice and therefore affecting the properties of the graphene as electrical mobility is reduced [13]. Non-covalent functionalization methods can also be used, which do not disrupt the sp^2^ bonding in graphene. This leaves the bonding structure of graphene intact as it involves electrostatic interactions, π-π stacking or van der Waals interactions [12]. High carrier mobility and gate tunability is desirable for high sensitivity of graphene sensors. It is therefore preferable that functionalization methods have minimal disruption on the crystal lattice of graphene and its associated electronic transport properties [14], with a functional layer as thin a layer as possible, in order to maintain the proximity of the graphene layer to the sensing system [15]. Noncovalent functionalization is thus advantageous in the manufacture of graphene field-effect transistor (GFET) or resistor-based sensors. Much research is currently ongoing into the use of GFET or resistor based sensors using varied functionalization techniques and for the detection of a wide range of analytes including real-time monitoring of insulin [16], the detection of human chorionic gonadotropin as a cancer risk biomarker [17], carcinoembryonic antigen [18], and drug quantification [19].

Functionalization allows for the availability of binding moieties e.g., amine groups, which in turn allow for further bio-functionalization via crosslinking chemistries such as carbodiimide EDC(*N*-(3-Dimethylaminopropyl)-*N′*-ethylcarbodiimide hydrochloride)-NHS(*N*-Hydroxysuccinimide). Such non-covalent functionalization requires that molecules have π-conjugated systems which overlap the π orbitals of the graphene, e.g., aromatic compounds [20]. One example of a common aromatic amine compound used to this end in biosensor development is aniline, which has been used in biosensing for such applications as the electrochemical detection of human chorionic gonadotropin using graphene screen printed electrodes [21], detection of neutrophil gelatinase-associated lipocalin using graphene/polymerized aniline nanocomposites [22] and real-time detection of ammonia using graphene/polymerized aniline nanocomposite films [23]. Another example of an aromatic amine compound used in biosensor development is 1,5-diaminonaphthalene (DAN) which has been previously used for the electrochemical detection of chloramphenicol using edge plane pyrolytic graphite sensors [24] and the detection of sulfamethoxazole using glassy carbon electrodes [25]. However DAN has also been realized as a functionalization method for use in GFET or resistor based sensors, for example in the detection of Hg^2+^ [26] or for hydrogen gas sensing [27]. Much research into the use of DAN for graphene functionalization has been demonstrated when utilized in its polymeric form (pDAN), for the creation of thin conducting films on top of the graphene surface for sensing purposes, including the use of pDAN films to improve enzymatic electrochemical sensing [28] and for the detection of lactose [29]. Monomeric DAN, however, can also be used in non-covalent functionalization via π-π stacking onto graphene surfaces, which can be achieved by a simple drop-cast technique. DAN orients flat on the graphene surface due to π-π stacking interactions between the naphthalene of the DAN molecule and the aromatic structure of the grapheme [27], thus offering a simple route for functionalization.

Our recent work on real-time analysis of graphene GFETs showed that graphene surfaces are extremely sensitive to the reagents used in surface modification, e.g., water and ethanol [5]. Although such simple drop-cast methods for non-covalent binding are advantageous with respect to avoidance of exposure of graphene surfaces to harsh chemicals in more aggressive functionalization processes (electropolymerization, covalent functionalization etc.), it is important to explore the stability of such drop-cast layers. Preservation of graphene’s electrical properties during functionalization is essential but the potential variable nature of direct absorption of biomolecule receptors on to the graphene surface means chemical functionalization before bio-functionalization is a pre-requisite step in biosensor development. In this work, graphene surfaces were functionalized with 1,5-DAN via a drop-cast method, leading to π-π stacking of the DAN molecules onto the graphene surface. The stability of the DAN layer on graphene and that of the subsequent attachment of a monoclonal IgG (model) antibody was explored following multiple wash steps, using X-ray photoelectron spectroscopy (XPS), Raman analysis, and real-time resistance measurements. The viability of drop-cast DAN as a simple and quickly applied functionalization layer for use in biosensor development is investigated.

## 2. Materials and Methods

### 2.1. Materials

Graphenea (Cambridge, MA, USA): Monolayer graphene on 300 nm thermal oxide SiO_2_/Si wafers. DOW Electronics Materials (Portland, ME, USA): Microchem LOR 3A positive photoresist; Microposit S1805 G2 Positive resist; Microposit MF-CD-26 developer and Microposit Remover 1165. Sun Chemical Corporation (Parsippany, NJ, USA): Dielectric paste. Fisher Scientific UK Ltd. (Loughborough, Leicestershire, UK): Phosphate buffered saline (PBS) containing 0.01 M phosphate, 0.0027 M KCl, and 0.137 M NaCl, pH 7.4. Metrohm UK Ltd. (Runcorn, Cheshire, UK): Dropsense Graphene modified screen-printed electrodes. Sigma Aldrich Company Ltd. (Gillingham, Dorset, UK): 1,5-diaminonaphthalene, Bovine Serum Albumin (BSA) and all other reagents (analytical grade). Hytest Ltd. (Turku, Finland Proper, Finland): Monoclonal anti-HBsAg (IgG).

### 2.2. Methods

#### 2.2.1. Graphene Device Manufacture

The graphene resistor devices used in this work were fabricated using CVD (chemical vapor deposited) graphene, transferred on to SiO_2_/Si wafers. Devices were manufactured and passivated according to methods outlined in our previous work [5].

#### 2.2.2. Functionalization of Graphene

DAN functionalization: 10 mM DAN solution diluted in 70% ethanol was drop-cast onto the graphene devices (20 µL) and incubated at room temperature (RT) for 2 h. The droplet was topped up during the 2 h incubation to avoid evaporation and drying out of the solution which could affect the results. Devices were subsequently washed in ethanol, followed by DI (de-ionized) water and gently dried with N_2_.

Antibody attachment: Solution prepared as follows: final concentration of antibody (monoclonal IgG model system antibody) = 0.1 mg/mL: 1.4 µL Neat Ab + 6 µL EDC (100 mM) + 15 µL NHS (100 mM) + 77.6 µL PBS (Order of addition: PBS > Ab > EDC + 5 min > NHS + 10 min). Activated antibody + EDC/NHS solution (15 µL) was drop-cast onto the DAN functionalized graphene devices and left to incubate at RT for 2 h (intermittent agitation throughout). Devices were washed with 1 × PBS (pH 7.4), followed by DI water and gently dried with N_2_.

Blocker attachment: 1% BSA blocker diluted in 1 × PBS (pH 7.4), drop-cast onto the Ab/EDC-NHS/DAN functionalized graphene devices, and was incubated for 30 min at RT. Devices were washed with 1 × PBS (pH 7.4), followed by DI water and gently dried with N_2_.

#### 2.2.3. Electrical Measurements

Real-time resistance measurements: chips consisting of three CVD graphene resistor devices on a SiO_2_/Si substrate were used for real-time resistance measurements. One graphene resistor was measured at a time using a standard lock-in technique under ambient conditions (temperature 20 °C, normal atmospheric pressure). In a current-fixed regime, currents of 0.1 or 1.0 µA were passed through the resistor devices. In a voltage-fixed regime, fixed voltages of 0.1 or 4 mV were applied across the graphene resistor device. Obtained resistance values were insensitive to the measurement regime used. Typical device resistance linearity over a 0.5 μA–5 μA current range is shown in Figure S6.

## 3. Results and Discussion

### 3.1. Cyclic Voltammetry

**Figure 1:**
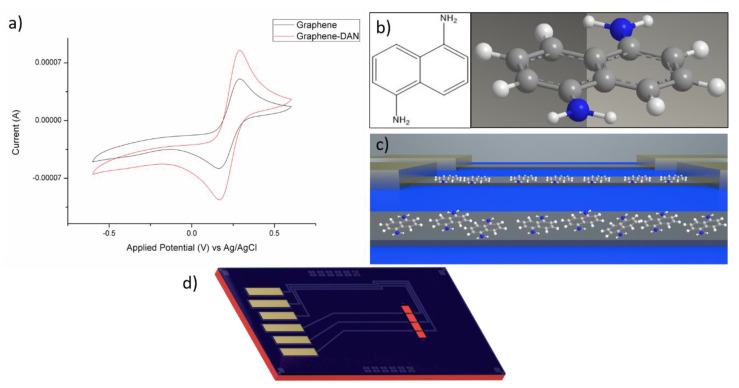
(**a**) Cyclic voltammograms (CVs) of a graphene screen-printed electrode before and after surface modification with 1,5-diaminonaphthalene (DAN): unmodified graphene (black) and DAN modified (red). CVs were carried out in [Fe(CN)_6_]^−3^/[Fe(CN)_6_]^−4^ in 1 × PBS (pH 7.4), at a scan rate of 50 mV/s and a potential window of −0.6–0.6 V; (**b**) structure of DAN; (**c**) illustration of DAN on graphene resistor devices; (**d**) Schematic of graphene resistor chips.

Surface modification steps were monitored using cyclic voltammetry, using an Autolab PGSTAT302N potentiostat (Metrohm, Runcorn, Cheshire, UK), [30,31] (Figure 1a) in 5 mM [Fe(CN)_6_]^−3^/^−4^ in 1 × PBS (pH 7.4), at a scan rate of 50 mV/s. An increase in peak current is observed after DAN functionalization (Figure 1a). This synergistic effect has been observed in several studies involving pDAN films on carbon surfaces [25,32]. The mechanisms for this are still not fully understood [29] but have been attributed in literature to the high specific surface area, electrical conductivity, easier electron transfer, which can be attributed to favorable electrostatic interactions between the negatively charged redox probe and the positively charged amine groups [33], and π-π interactions between the pDAN layer and the graphene surface [32,34]. Drop-cast monomeric DAN, therefore, shows a similar synergistic effect of increased peak current when used as a functionalization layer on carbon surfaces, to that seen with pDAN functionalization layers. The surface area of the electrodes was calculated using Randle-Sevcik Equation [35]. The area of the blank electrode was found to be 0.059 cm^2^, and for the DAN modified electrode, the surface area was 0.097 cm^2^.

To ensure that the measured peak current changes were due to the presence of DAN on the graphene surface and not due to solvent interference, a control sample was measured using ethanol only in the place of DAN (using the same drop-cast method). A cyclic voltammogram for the ethanol only control can be seen in Figure S1, no significant increase in peak current is observed, confirming therefore that changes in peak currents are not due to solvent effects on the graphene surface and thus suggesting that DAN is present on the graphene surface and actively involved in the electron transfer process. The presence of amine groups on a carbon surface after drop-cast DAN modification was investigated using fluorescent microscopy and the resultant images can be found in Figure S4.

### 3.2. Surface Characterization-Raman Spectroscopy

**Figure 2:**
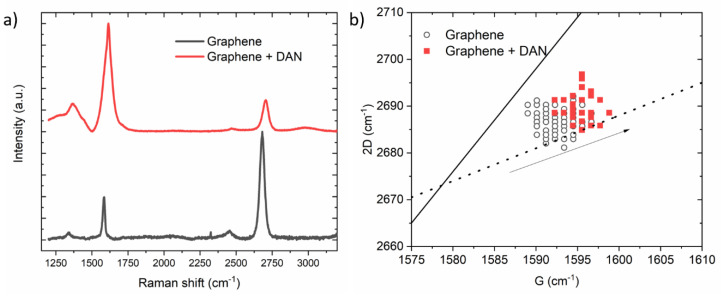
(**a**) Raman spectra for an unmodified graphene device (black curve) and a graphene device functionalized with DAN (red curve); (**b**) Scatter plot marking G and 2D positions from an unmodified graphene device (white circles), and after modification with DAN (red squares).

Raman spectroscopy was used to monitor the effects of surface modification of monolayer graphene with drop-cast DAN. Extended Raman scans were taken using a Renishaw inVia Raman system (Renishaw, Wotton-under-Edge, Gloucestershire, UK) with a 532 nm laser. Before surface modification, typical Raman spectra of graphene devices were obtained. Representative point spectra are shown in Figure 2a. Following surface modification with DAN (described in Section 2.2.2), changes in the Raman spectra were consistent with the addition of a small aromatic molecule to the surface of graphene (Figure 2a). The defect-related “D” peak of graphene at ~1350 cm^−1^ can vary across the surface of the graphene device before modification, however, its intensity ratio to the G peak (*I*_D_*/I*_G_) is generally seen to increase after modification with DAN (i.e., from ~0.1 to ~0.2). The full width half maximum (FWHM) of the “D” peak broadens from ~34 cm^−1^ to ~190 cm^−1^ after surface modification. This broadening of the peak suggests that it contains a component from the C-N band at 1247 cm^−1^, from the DAN, as further evidenced by its asymmetric shape. The intensity ratio of the “2D” peak (~2678 cm^−1^) to the G peak changes dramatically following DAN modification, from *I_2D_/I_G_* ~2.5 for unmodified graphene to ~0.3 following modification. This change in peak ratio is consistent with the addition of aromatic carbon-containing molecules to monolayer graphene surfaces [36,37].

The positions of the G and 2D peaks can be used to indicate doping and strain in graphene. Raman spectra were obtained, centered at 1600 cm^−1^ and 2600 cm^−1^, in a grid pattern. Peak analysis based on previous reports [36,38,39] was performed using custom scripts. Graphene on SiO_2_ is typically p-doped [40], under ambient conditions, this is in contrast to high vacuum, where the graphene (on SiO_2_) might be intrinsic or n-doped [41]. A shift in the direction of the arrow in Figure 2b indicates electron donation from DAN [36]. Before modification, the position of the 2D/G peaks are consistent with the p-doping arising from the SiO_2_ substrate, Figure 2b, (white circles), compared with values reported in the literature (plotted guidelines from literature values—solid line—charge screening, dashed line-doping). After surface modification, the resulting points (Figure 2b, red squares) are consistent with a shift towards the charge neutrality point—following surface modification with DAN, which partially neutralizes the p-doping effect of the substrate [42]. No statistically significant indication of the underlying graphene undergoing strain was measured, consistent with a conformal coating of the DAN layer on the graphene substrate with no evidence of unwanted mechanical effects. It should be noted that the resultant graphene + DAN spectra also contain peak components from both the graphene substrate and the attached DAN molecules, which may complicate the analysis and preclude this method from providing an accurate estimate of the doping levels in graphene, which is therefore presented as an overall trend.

### 3.3. Surface Characterization—X-ray Photoelectron Spectroscopy (XPS)

**Figure 3:**
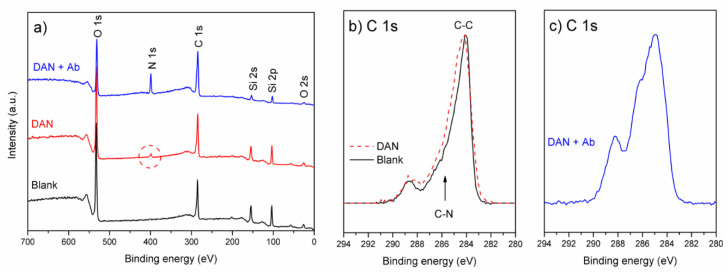
XPS spectra of successively functionalized graphene on SiO_2_: (**a**) Survey spectra showing the appearance of N 1s signal following surface modification with DAN and screening of Si substrate signal; (**b**) comparison of normalized C 1s peak for blank graphene and DAN modified samples showing broadening of C-C component/increase in the region associated with C-N components; (**c**) a significant change in the shape of normalized C 1s signal following attachment of antibody.

Functionalization with amine moieties should further allow for attachment of receptor molecules in affinity biosensors, e.g., antibody molecules. To that end, non-covalent functionalization of graphene with drop-cast DAN has been used for attachment of an antibody (monoclonal IgG model system antibody), providing evidence of its viability as a functional layer. X-ray photoelectron spectroscopy (XPS) measurements were performed, on unmodified graphene (before surface modification with DAN (blank graphene)) on SiO_2_/Si substrates, graphene with surface modification (DAN) and graphene with surface modification followed by attachment of an antibody (DAN + Ab), using a Kratos Axis Supra XPS system (Kratos Analytical, Wharfside, Manchester, UK) using an Al Kα monochromatic X-ray source with an emission current of 15 mA at 20 eV pass energy. A minimum of three scan locations per sample were used to ensure the spectra were representative of the surface. Figure 3a shows survey spectra comparing the O 1s, N 1s, C 1s and Si 2s/2p peaks. The unmodified/blank sample shows the expected C 1s and Si peaks associated with the graphene and substrate, along with an O 1s signal containing contributions from the thermal SiO_2_ layer along with organic contaminants on the graphene surface (C-O/C=O) [43]. Following the deposition of DAN, the appearance of an N 1s signal is seen (Figure 3a, circled) at a binding energy of around 400 eV (C-NH_2_), corresponding to an atomic concentration of approximately 2.2%. Following the application of the antibody (and after standard wash steps), a further increase in the N 1s signal is evident (10.9 at. %), along with an attenuation of the Si 2s/2p and O 1s substrate signals, consistent with surface antibody coverage. The atomic concentrations for the samples are summarized in Table 1.

The normalized C 1s core-level signal following background subtraction is shown in Figure 3b for the blank graphene, DAN and DAN + Ab samples. A relative increase in the C 1s signal around 285.6–285.9 eV (C-N contribution from the amine moiety) [44] and/or broadening of the sp^2^/sp^3^ C-C contribution above the predominantly sp^2^ graphene blank (Figure 3b) is consistent with attachment of the DAN molecule. Although little variation in C 1s intensity between samples is seen in the survey spectra, Figure 3c reveals a change in the components comprising the C 1s signal, with a significant relative increase in components commonly associated with C-O/C-N and C=O, as expected following attachment of the antibody and screening of the sp^2^ C-C signal, therefore indicating that drop-cast DAN introduces amine moieties to the graphene surface which subsequently allowed for the attachment of antibody receptors, showing the viability of drop-cast DAN as an amine functionalization method. Standard biosensor process incubation and wash steps were carried out with the results indicating that both the DAN and the subsequent attached antibody were still present on the graphene surface, demonstrating the functionalization was robust to standard biosensor processing steps.

### 3.4. Electrical Measurements

**Figure 4:**
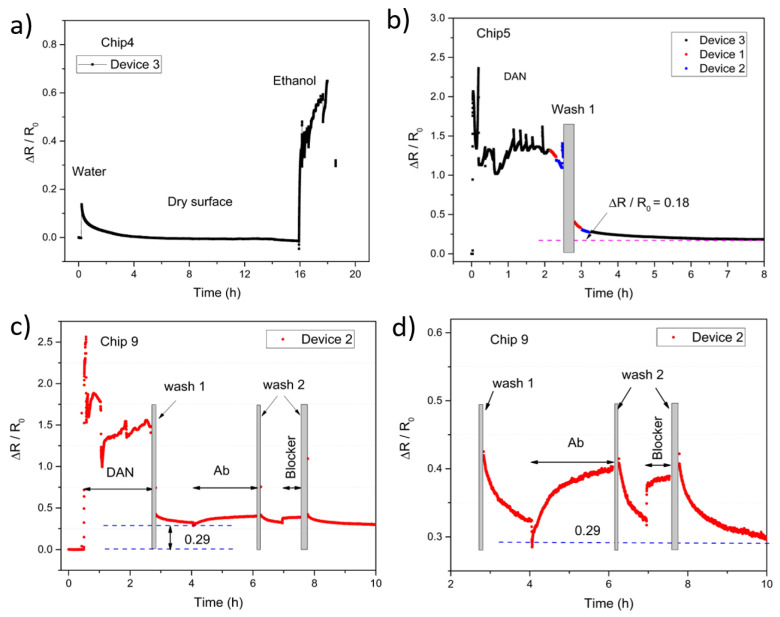
Real-time resistance measurements of the graphene functionalization process. Where ∆*R* = *R*_Device_ − *R*_0_, and *R*_0_ is the intrinsic device resistance. (**a**) Control experiment: Washed with DI water, left over-night, followed by the addition of 20 µL of 70% ethanol. (**b**) 2 h DAN incubation at RT, signal spikes due to topping up of the droplet to avoid evaporation of the DAN during incubation, followed by wash 1 (wash 1 = wash with ethanol, followed by DI water and gently dried with N_2_). (**c**) and (**d**) DAN incubation followed by wash 1, subsequent bio-functionalization stages (antibody and blocker incubations), followed by wash 2 (wash 2 = wash with 1 × PBS (pH 7.4), followed by DI water and gently dried with N_2_).

While graphene’s high sensitivity to environmental changes and surface modifications are extremely desirable in biosensing applications, these qualities also mean it is highly sensitive to entities other than the target analyte, e.g., buffer solutions, water or other solvents [5]. Figure 4a shows real-time monitoring of resistance changes of unmodified graphene in the presence of DI water and ethanol (used for dilution during drop-cast DAN functionalization), run as a control experiment to show the reaction of the graphene to the solvent components. The relaxation curve of the graphene washed with DI water, and allowed to dry and relax over-night, shows a relaxation time of approximately 4 h, a relaxation time is usual for real-time measurement of graphene that has been exposed to solution and is left to relax under ambient conditions. The relaxation may be associated with charge redistribution between charges related to surface absorbates on the graphene and those related to the SiO_2_/Si substrate [5,44]. It is therefore essential when determining resistance changes due to surface functionalization, to allow full relaxation to occur, before observing the final resistance change. An image of a passivated chip in the Biovici “sensor-Connect” connector (Biovici Ltd., Swansea, City and County of Swansea, UK) can be found in Figure S5.

Real-time resistance data, Figure 4b, shows an increase in resistance when DAN (in 70% ethanol) is placed on the graphene device surface. A 2 h incubation was used; however, it can be seen from the real-time results that saturation occurred after approximately 1 h. Spikes in resistance are visible during the 2 h DAN incubation, these are due to topping up of the solution to prevent evaporation and drying out of the sample. After washing (wash 1 = ethanol, followed by DI water and gentle drying with N_2_), relaxation occurred over 5.5 h, this relaxation time occurs after any exposure of the graphene to solution and it is not a DAN specific effect. Long relaxation times occur with solution on bare graphene, e.g., Figure 4a and after functionalization using solutions and wash steps, e.g., Figure 4b–d. These relaxation times also occur when graphene is functionalized by other methods, e.g., oligonucleotides and AuNPs [5]. The final resistance (after relaxation) is higher (an increase of 18%) than the bare (dry intrinsic) graphene resistance, indicating DAN is present on the surface (further device repeats can be found in the Supplementary Information Figure S2). This increase in resistance can be attributed to the shift toward the charge neutrality point of the graphene by DAN, consistent with the Raman results in Section 3.2. DAN is an electron-donating molecule, resulting in electron donation to graphene, which partially neutralizes the p-doping from SiO_2_/Si substrates [45,46] and therefore an increase in final resistance after DAN modification is consistent with this.

Figure 4c shows real-time resistance data for each surface modification stage. An increase in resistance is again seen during DAN functionalization, followed by relaxation (after wash 1), this time with a final resistance increase of 29% compared to dry intrinsic graphene resistance. Again, it is important to investigate the ability of the drop-cast DAN to provide amine moieties for further bio-functionalization with bioreceptors such as antibody molecules. The DAN functionalized graphene resistor devices were further modified via carbodiimide crosslinking of the amine groups of the DAN on the graphene surface with the carboxyl groups of the antibody molecules via EDC/NHS. Subsequent increases in resistance followed by relaxation (after wash 2 (wash 2 = 1 × PBS, followed by DI water and gentle drying with N_2_)) steps are also shown in Figure 4c,d for the antibody and blocker stages respectively (repeats can be found in the Supplementary Information Figure S3), a blocker is required to reduce non-specific binding and/or interaction of molecules with the sensor surface especially when testing in complex matrices such as plasma or serum. The final resistance increase, after antibody attachment and following the wash steps involved in the crosslinking/attachment processes, demonstrates a suitable and robust non-covalent amine functionalization method using drop-cast DAN, towards affinity biosensor development.

As there is a resistance change after surface modification (as shown in Figure 4), it is important to ascertain that these changes are in fact due to attachment of the desired DAN functionalization molecule and not due to solvent effects. I–V (current-voltage linear sweep) measurements of ethanol only on graphene resistor devices were carried out to show the effect of ethanol on the resistance of the graphene device. Graphene resistor devices were incubated with either 10 mM DAN solution or ethanol only solution for 2 h before being washed (wash 1) and gently dried with N_2_. The I–V linear sweep measurements were therefore performed on dry samples. The ∆*R/R*_0_ for DAN functionalized resistor devices was + 0.078 ± 0.039 (SD of N = 13), and the ∆*R/R*_0_ for the ethanol only control resistor devices was −0.069 ± 0.043 (SD of N = 13), individual measurements can be found in Supplementary Table S1. Ethanol has a slight p-type doping effect on graphene on SiO_2_ substrates [47], this p-doping effect can be attributed to structural re-arrangement during immersion or trace amounts of ethanol or impurities left on the surface after drying [48]. Increases in final resistance after functionalization can, therefore, be an indication of DAN attachment to a graphene surface (on SiO_2_/Si substrates). DAN is diluted in 70% ethanol, which has a slight p-doping effect; however, the final resistances increased above bare graphene (dry intrinsic resistance), meaning these changes in resistance are due to the likely electron donation from DAN and not due to solvent effects.

Variation in the final ∆*R/R*_0_ after DAN modification was observed in the electrical measurements. A possible reason for this could be attributed to levels of graphene contamination. Multiple sources of contamination can be present on a graphene device (surface) which could affect device-to-device performance issues. Amorphous carbon made and deposited on the graphene during the growth process (CVD), residues left by fabrication chemicals or residual polymer left behind from the graphene transfer process [49] can all add contamination cumulatively throughout manufacture. Device-to-device variations in graphene devices is an ongoing field-wide issue for biosensor development [50]. Causes can be both intrinsic, e.g., grain boundaries in CVD grown graphene and extrinsic, e.g., including polymer and resist residues [51]. The presence of resist contamination can affect the functionalization of the graphene with a desired functional molecule, leading to incomplete/patchy and varying coverage, adding to extrinsic device-to-device variation. Certain types of resist contain π-conjugated aromatic molecules and as a result, bind to the graphene surface via π-π stacking interactions. These components can include novolac resin, diazonapthoquinone (DNQ) and cresol [52] and can affect the amount of desired functionalization molecules able to attach to the graphene surface. This is a likely cause of the variation seen in the ∆*R*/*R*_0_ of DAN functionalized graphene resistor devices. Whether the contaminants e.g., aromatic resist components, are electron acceptors or donors can affect doping of the graphene, and as the level of contamination varies with each manufacturing step, the level of doping will, therefore, show variation between batches but also between devices in the same batch.

### 3.5. Investigation of Wash Steps

Exposure of the functionalization layer to multiple solvents is inevitable during affinity biosensor development, it is, therefore, essential to check the ability of the drop-cast DAN to withstand these multiple solvent exposures. The strength of the π-π stacking between the graphene surface and the aromatic molecules of the functionalization layer e.g., DAN can make the surface stacking quite stable against rinsing or other solution processing [53]. An investigation was carried out using XPS and Raman to determine the robustness of π-π stacked DAN (drop-cast) attachment to graphene against sequential wash steps that may be necessary for subsequent bio-functionalization steps of biosensor fabrication.

The wash step investigation consisted of an initial ethanol wash to remove residual DAN from the surface (carried out for all samples), followed by 1, 2, or 3 DI water washes, to simulate further standard biosensor process steps. XPS analysis indicates that the DAN layer is not affected or removed by sequential wash steps. This is shown in Table 2, where the atomic concentration of nitrogen, as calculated from the N 1s region at 400 eV, remains consistent between each of the different sequential wash steps and therefore indicates that the amine groups of the DAN molecules are still present on the graphene surface. The concentration of oxygen and carbon show more variation compared to the nitrogen, this may be partly due to surface contaminants or defects that are present on the graphene surface and is unlikely to be directly correlated with the DAN attachment [54].

**Figure 5:**
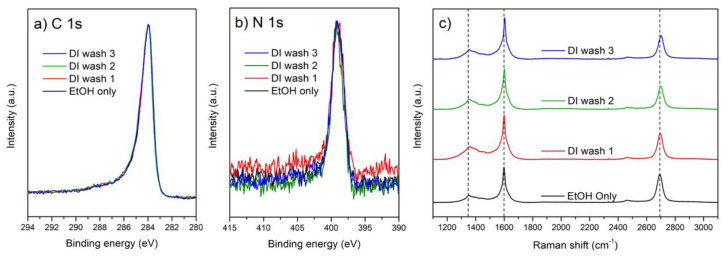
XPS characterization of CVD graphene before and after DAN functionalization with (**a**) C 1s and (**b**) N 1s XPS spectra. (**c**) Raman spectroscopy of CVD graphene before and after DAN functionalization. All samples modified with DAN and the following wash procedures applied, black = ethanol wash only, red = ethanol wash followed by 1 DI water wash, green = ethanol wash followed by 2 sequential DI water washes, blue = ethanol wash followed by 3 sequential water washes.

After peak fitting, several additional peaks were found in the unmodified graphene sample at ~284.76, ~285.67, and ~286.27 eV, Figure 5a, corresponding to sp^3^ carbon, C-O and C=O bonds [43]. These bonds can appear due to defects in the graphene structure and trace PMMA (Polymethyl methacrylate) contaminants on the graphene surface, from the transfer of the graphene from its copper catalyst to the SiO_2_/Si substrate [53,55]. After surface modification with DAN, additional peaks appear at ~285.7 eV and ~287.2 eV respectively. These correspond to the C-N and C=N bonds which are in agreement with published literature [28,56]. Following the sequential wash steps, no changes in the C-N and C=N bond concentrations are observed in the carbon spectra, further supporting that the DAN layer is unaffected by the wash procedures. Figure 5b shows the nitrogen spectra with two peaks present after surface modification, at ~399.1 eV and ~401.45 eV, these peaks are associated with C-N and C-N + bonds, respectively [54,57].

Raman spectra were also obtained following subsequent wash steps. Figure 5c shows the D-peak intensity for unmodified samples with a significant increase after surface modification with DAN observed. After surface modification and the initial ethanol wash, the D-peak, G-peak, and 2D-peak shift to 1351 cm^−1^, 1598 cm^−1^, and 2692 cm^−1^, respectively. After each successive DI water wash, the peaks red shift slightly until, after 3 DI water washes, the D-peak, G-peak, and 2D-peaks reach 1358 cm^−1^, 1603 cm^−^^1^, and 2700 cm^−1^, respectively. These shifts are consistent with graphene being p-doped with water [58]. Regardless of the number of wash steps, the intensity ratios between the D-peaks and G-peaks (*I*_D_*/I*_G_), at ~1350 cm^−1^ and 1580 cm^−1^ respectively, are consistently around 0.2. Also, the *I*_2D_*/I*_G_ ratio decreases from approximately 2 to approximately 0.55 for the modified samples, which is consistent for all wash steps. Both XPS and Raman results from subsequent wash steps of drop-cast DAN modified graphene demonstrate that the functionalized surface is robust for use in a biosensor process sequence after multiple solvent exposures.

## 4. Conclusions

Drop-cast DAN as a robust and facile method to amine functionalize carbon surfaces has been demonstrated. The addition of a functional layer containing amine groups allows for further bio-functionalization with specific receptors, using common crosslinking chemistries such as carbodiimide EDC-NHS, for the development of affinity biosensors. Real-time resistance measurements, cyclic voltammetry, XPS, and Raman analysis showed successful modification of graphene surfaces with DAN. As the purpose of surface modification with DAN was to provide amine moieties for further bio-functionalization with receptor molecules, the successful attachment of an antibody molecule was also confirmed, further demonstrating the viability of drop-cast DAN as a surface functionalization strategy. The robustness of the DAN and subsequently crosslinked antibody to withstand multiple washes was also demonstrated, with the presence of the DAN layer and successful antibody attachment confirmed via XPS, Raman spectroscopy, cyclic voltammetry, and real-time resistance measurements. The viability of drop-cast DAN as a surface functionalization method, providing available amine moieties was demonstrated with the resultant attachment shown to withstand multiple wash steps, therefore, providing a quick and simple route to prototype for biosensor development.

## Figures and Tables

**Table 1:** Atomic concentrations derived from O 1s, N 1s, C 1s and Si 2p regions obtained via XPS of successive antibody functionalization steps.

Sample	O 1s	N 1s	C 1s	Si 2p
Blank Graphene	36.0 ± 0.4%	–	45.6 ± 1.1%	18.4 ± 1.5%
DAN	30.9 ± 1.9%	2.2 ± 0.1%	52.3 ± 3.7%	14.5 ± 1.8%
DAN + Ab	20.4 ± 0.1%	10.9 ± 0.1%	62.8 ± 0.1%	5.9 ± 0.1%

**Table 2:** The Atomic concentration of oxygen (O 1s), nitrogen (N 1s) and carbon (C 1s) via XPS for DAN modified graphene samples after multiple wash steps.

Sample	Element	Binding Energy Position (eV)	Atomic Concentration (%)
Blank Graphene (Gr)	O 1s	532.22	61.83 ± 0.32
	N 1s	N/A	N/A
	C 1s	284.02	38.17 ± 0.32
Gr + EtOH Wash Only	O 1s	532.10	50.94 ± 0.35
	N 1s	399.30	2.56 ± 0.18
	C 1s	284.03	46.50 ± 0.36
Gr + EtOH Wash + 1 DI Water Wash	O 1s	531.90	48.60 ± 0.33
	N 1s	398.70	2.59 ± 0.15
	C 1s	284.00	48.81 ± 0.34
Gr + EtOH Wash + 2 DI Water Wash	O 1s	532.00	46.04 ± 0.34
	N 1s	399.30	2.69 ± 0.14
	C 1s	283.98	51.27 ± 0.35
Gr + EtOH Wash + 3 DI Water Wash	O 1s	531.90	46.52 ± 0.34
	N 1s	399.20	2.60 ± 0.17
	C 1s	283.99	50.88 ± 0.35

## Supplementary Materials

The following are available online at https://www.mdpi.com/2079-4991/10/9/1808/s1, Figure S1: Cyclic Voltammograms of bare, DAN functionalized and ethanol control. Figure S2: Real-time functionalization results, Figure S3: Real-time functionalization process stages. Figure S4: Fluorescent microscopy images. Figure S5: Image of a passivated chip in the Biovici “sensor-Connect” connector, Figure S6: Typical channel resistance linearity over the 0.5 μA–5 μA current range, Table S1: ∆*R*/*R*_0_ for graphene devices immersed in ethanol or DAN.
