# Supplementary material for: A Facile Method for the Non-Covalent Amine Functionalization of Carbon-Based Surfaces for Use in Biosensor Development
Source: Nanomaterials (Basel). 2020 Sep 10;10(9):1808. doi: 10.3390/nano10091808 (PMC7559712; doi:10.3390/nano10091808)
Supplement: Supplementary file 1 [file nanomaterials-10-01808-s001.pdf]

nanomaterials

Supplementary Materials

A Facile Method for the Non-Covalent Amine Functionalization of Carbon-Based Surfaces for Use in Biosensor Development

Ffion Walters [1,*], Muhammad Munem Ali [1], Gregory Burwell [2], Sergiy Rozhko [3], Zari Tehrani [1], Ehsaneh Daghigh Ahmadi [1], Jon E. Evans [1], Hina Y. Abbasi [1], Ryan Bigham [2], Jacob John Mitchell [1], Olga Kazakova [3], Anitha Devadoss [1,*] and Owen J. Guy [1,4,*]

[1] Centre for NanoHealth, College of Engineering, Swansea University, Swansea SA2 8PP, UK; 823439@swansea.ac.uk (M.M.A.); z.tehrani@swansea.ac.uk (Z.T.); e.daghighahmadi@swansea.ac.uk (E.D.); j.e.evans@swansea.ac.uk (J.E.E.); h.y.abbasi@swansea.ac.uk (H.Y.A.); j.j.mitchell@swansea.ac.uk (J.J.M.)
[2] Department of Physics, College of Science, Swansea University, Swansea SA2 8PP, UK; g.burwell@swansea.ac.uk (G.B.); r.m.bigham@swansea.ac.uk (R.B.)
[3] National Physical Laboratory, Quantum Metrology Institute, Teddington, Middlesex TW11 0LW, UK; sergiy.rozhko@npl.co.uk (S.R.); olga.kazakova@npl.co.uk (O.K.)
[4] Department of Chemistry, College of Science, Swansea University, Swansea SA2 8PP, UK
* Correspondence: 968191@swansea.ac.uk (F.W.); anitha.devadoss@swansea.ac.uk (A.D.); o.j.guy@swansea.ac.uk (O.J.G.); Tel.: +44-(01)-792602985 (F.W.); +44-(01)-792606475 (A.D.); +44-(01)-792513181 (O.J.G.)

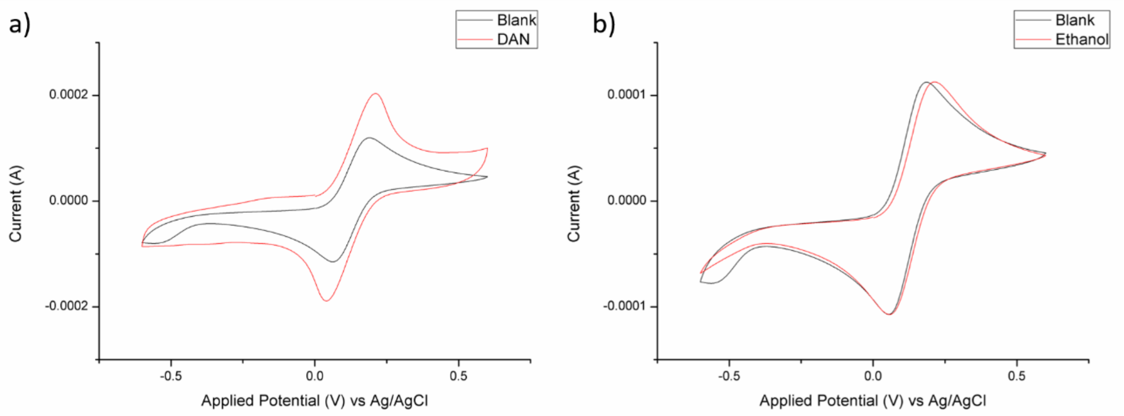

Figure S1. (**a**) Cyclic voltammograms of a bare graphene (blue) and DAN functionalized (red); (**b**) Cyclic voltammograms of a bare graphene (blue) and DAN functionalized (orange). CVs were carried out in $[Fe(CN)_6]^{-3}/[Fe(CN)_6]^{-4}$ in 1 × PBS pH 7.4, at a scan rate of 50 mV/s and a potential window of −0.6–0.6 V.

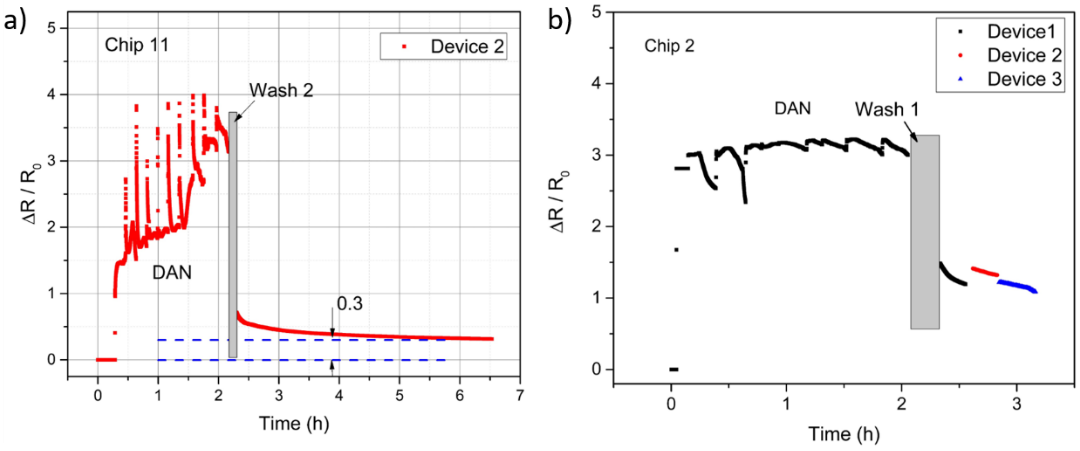

Figure S2. Real-time resistance measurements of the DAN functionalization process. Where $\Delta R = R_{Device} - R_0$, and R_0 is the initial resistance measurement. (**a**) 2 h DAN incubation at RT, followed by wash 1 and relaxation; (**b**) 2 h DAN incubation at RT, followed by wash 1, relaxation of all three resistors on the chip are indicated. Wash 1 = wash with DI water and gently dried with N_2. Wash 2 = wash with 1 x PBS (pH 7.4), followed by DI water and gently dried with N_2.

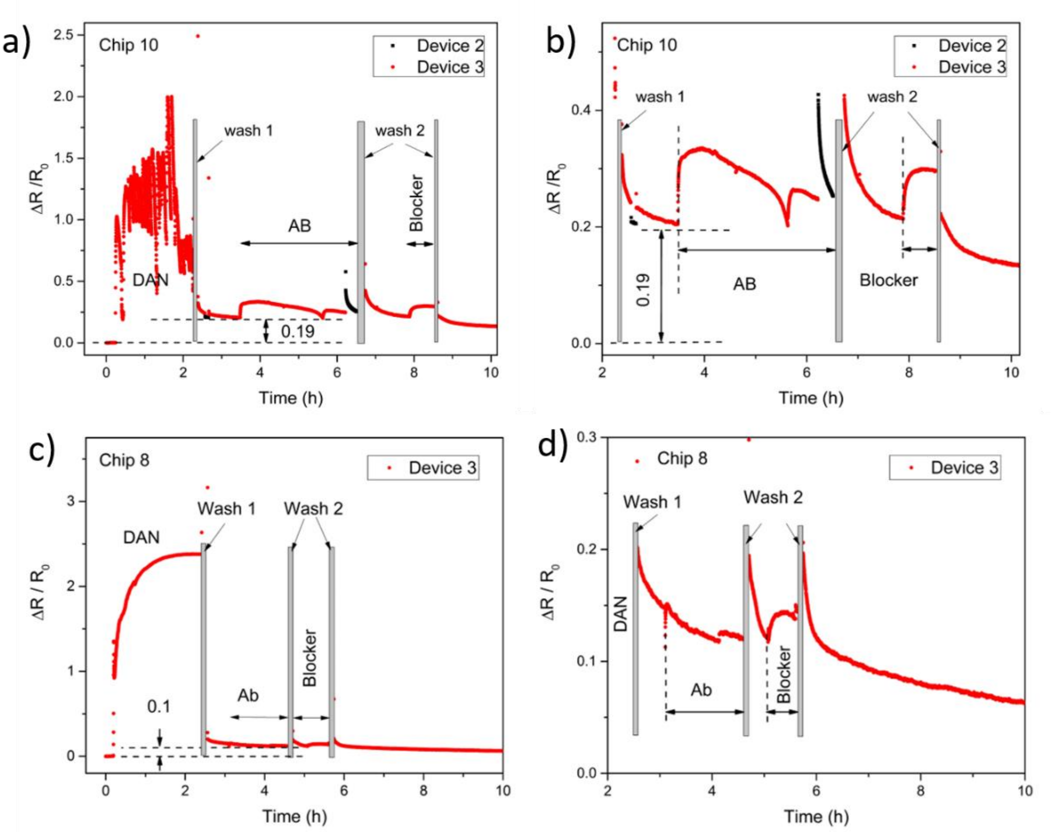

Figure S3. Real-time resistance measurements of the graphene functionalization process. Where $\Delta R = R_{Device} - R_0$, and R_0 is the intrinsic graphene resistance. (**a**) Functionalization stages (chip 10); (**b**) antibody and blocker stages (chips 10); (**c**) functionalization stages (chip 8); (**d**) antibody and blocker stages (chip 8). Wash 1 = wash with DI water and gently dried with N_2. Wash 2 = wash with 1 × PBS (pH 7.4), followed by DI water and gently dried with N_2.

Confirmation of the presence of amine groups at the graphene surface was investigated by via an amine reactive fluorescent dye. Following surface modification with drop cast DAN, HOPG (Highly oriented pyrolytic graphite) samples were incubated with 100 µl of amine-reactive fluorescent dye Alexa fluor 568. The dye specifically binds to amine terminated surfaces. The resulting fluorescence microscopy images are shown in Figure S4. As expected, the blank untreated surface showed no fluorescent properties (Figure S4a). However, the drop-cast DAN modified sample exhibited fluorescence following incubation with amine-reactive fluorescent dye Alexa fluor 568, confirming that amine groups are present on the HOPG surface after DAN modification.

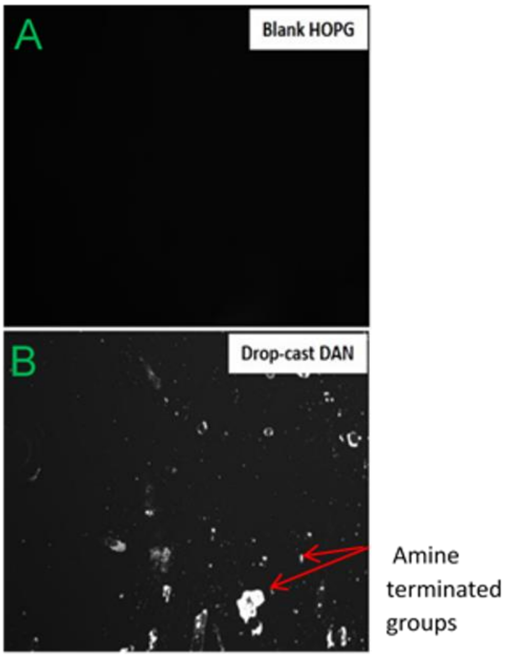

Figure S4. Fluorescence microscopy images taken after incubation of 100 µl drop of amine dye: (**a**) blank HOPG control, which exhibits no amine terminated surfaces; (**b**) drop-casted DAN sample, which exhibits amine terminated surfaces.

a)

Device Number	$\Delta R/R_0$ Ethanol only	
1	−0.04883	
2	−0.05041	
3	−0.04838	
4	−0.05375	
5	−0.05444	
6	−0.05640	
7	−0.06302	
8	−0.04917	
9	−0.21631	
10	−0.05772	
11	−0.07085	
12	−0.06543	
13	−0.06660	
	Mean	−0.06933
	SD	0.043019

b)

Device Number	$\Delta R/R_0$ 10 mM DAN	
14	0.10012	
15	0.07942	
16	0.05191	
17	0.05810	
18	0.07568	
19	0.06206	
20	0.08560	
21	0.01496	
22	0.00436	
23	0.15067	
24	0.12657	
25	0.10296	
26	0.10489	
	Mean	0.078253
	SD	0.039485

Table S1. (**a**) $\Delta R/R_0$ for graphene devices immersed in ethanol (2 h incubation), follow wash 1 (wash 1 = wash with ethanol, followed by DI water and gently dried with N_2); (**b**) $\Delta R/R_0$ for graphene devices immersed in 10 mM DAN solution (2 h incubation), follow wash 1 (wash 1 = wash with ethanol, followed by DI water and gently dried with N_2).

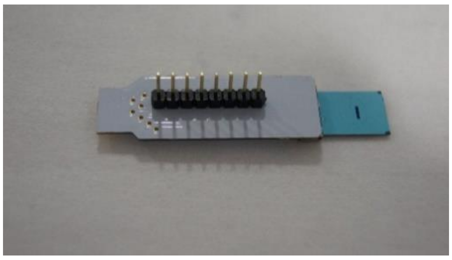

Figure S5. Image of a passivated chip in the Biovici "sensor-Connect" connector for real-time resistance measurements.

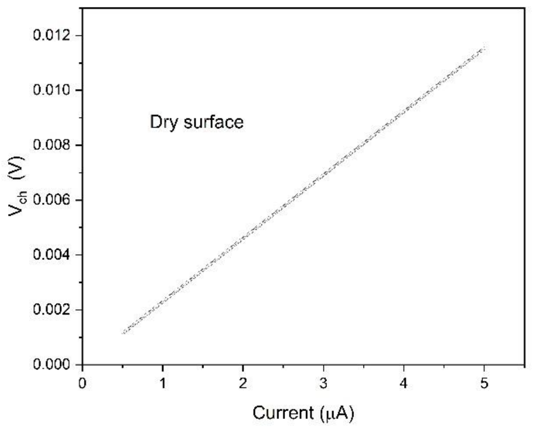

Figure S6. Typical channel resistance linearity over the 0.5 μA–5 μA current range. The I–V characteristics were measured by the four-pole method.
